# Patient understanding of moles and skin cancer, and factors influencing presentation in primary care: a qualitative study

**DOI:** 10.1186/1471-2296-11-62

**Published:** 2010-08-31

**Authors:** Fiona M Walter, Elka Humphrys, Simon Tso, Margaret Johnson, Simon Cohn

**Affiliations:** 1 General Practice & Primary Care Research Unit, Department of Public Health & Primary Care, University of Cambridge, Cambridge CB2 0SR, UK; 2 School of Primary, Aboriginal and Rural Health Care, University of Western Australia, Crawley, Western Australia; 3 University of Cambridge School of Clinical Medicine, Cambridge, CB2 0SP, UK; 4 Lay member

## Abstract

**Background:**

Melanoma incidence in the UK has doubled over two decades, yet there is conflicting evidence about factors which prompt or delay patients seeking advice. Aim: To explore patient understanding of pigmented skin lesions (moles) and skin cancer, and factors which influence seeking help in primary care.

**Method:**

Semi-structured interviews with forty MoleMate Trial participants, analysed using the theoretical framework of the Safer-Andersen model of Total Patient Delay.

**Results:**

Patient understanding and awareness was influenced by personal, family and friends' experiences of moles, skin cancer and other cancers, knowledge of risk factors, and the lay media. The route to consulting was complex and often iterative. For lesions that people could see, detecting and appraising change was influenced by comparisons with a normal mole on themselves, a family member, friend or image. Inferring illness came about with recognition of changes (particularly size) as serious, and associated 'internal' symptoms such as pain. For lesions that people could not see, family, friends and health professionals detected and appraised changes. Deciding to seek help was often prompted by another person or triggered by rapid or multiple changes in a mole. Three of four people subsequently diagnosed with melanoma did not seek help; instead, their GP opportunistically noticed the lesion.

**Conclusions:**

Changing moles are often perceived as trivial and not signifying possible skin cancer. This study contributes to current national strategies to improve patient awareness and earlier diagnosis of cancer by highlighting factors that can trigger or act as barriers to seeking help.

(ISRCTN79932379)

## Background

Malignant melanoma is the major cause of death from skin cancer in the UK. Over the last twenty years the incidence has more than doubled, with 9,500 new cases and 1,800 deaths in 2006 in England and Wales (age standardised incidence 13.7/100,000 people). The most important prognostic feature for patients with melanoma is the thickness of the primary tumour at diagnosis (thickness of 1 mm or less at diagnosis- 95% disease free after 5 years; thickness of 3 mm or more at diagnosis- 60% disease free after 5 years) [1]. Although the National Institute for Health and Clinical Excellence guidelines on skin cancer [2] and the recent National Awareness and Early Diagnosis Initiative for Cancer (Department of Health and Cancer Research UK, November 2008) advocate the raising of public awareness of symptoms and encouragement of people to seek help early, there is conflicting evidence about the impact of this on time from symptom awareness to presentation in primary care, and the thickness of the lesion at diagnosis [3,4]. However, it is likely that a shorter time from awareness to diagnosis will reduce psychological distress and may increase the proportion of thinner melanomas [5].

While patients frequently present to their general practitioners (GPs) with concerns about moles or other pigmented skin lesions, few will be diagnosed as melanoma: even among higher risk groups such as men aged over 60 years, less than 1 in 33,000 moles are estimated to become malignant [6]. A mole, or melanocytic naevus, is extremely common, with most people having between 5 and 20 moles which may vary in size, shape and colour. Moles may be congenital, but usually appear during childhood, and an increase in number is associated with age, fair skin and sunlight exposure. Patients need to be able to distinguish these 'normal' changes from 'abnormal' changes in size, shape or colour, which may indicate melanoma, in order to make the decision to seek help from their GP.

There is a growing literature concerning seeking help for cancer symptoms, as the time from symptom detection to presentation in primary care (known as 'patient delay'), and the time from presentation in primary care to referral to secondary care (known as 'primary care delay') are likely to be key determinants of patient outcomes for melanoma and other cancers [7]. A systematic review of patient delays in presenting with cancer symptoms suggested that fear of embarrassment (that the symptoms were trivial or affected a sensitive body area) or fear of cancer (pain, suffering and death) are major contributors to delay in diagnosis [8]. Older age, non-disclosure of symptoms, living alone, and negative attitudes towards the GP, can also influence delays in symptom presentation for breast cancer [9]. Misinterpretation of symptoms is another important factor contributing to patient delay in presenting with symptoms of upper gastro-intestinal [10], colorectal [11], and oral [12] cancer symptoms.

It is currently unknown how much these factors, or patient understanding of moles, influence seeking help for symptoms of skin cancer although factors which influence delay in the diagnosis of melanoma have been studied. There are other studies which suggest the utility of investigating these patient factors [13,14], however, most have used quantitative, retrospective methods of data collection from secondary care cohorts of people diagnosed with melanoma which limit the generalisibility of their findings to primary care populations with benign lesions as well as skin cancer. We therefore conducted interviews with the aim of exploring how patient understanding and evaluation of their moles influenced their decision to consult in primary care.

## Methods

Semi-structured telephone interviews were conducted between April and August 2008 with people who had been recruited to the feasibility phase of the MoleMate™ UK Trial. This randomised controlled trial (ISRCTN79932379, Cambridgeshire REC approval 07/H0308/167, approval also covered telephone interviews), funded by the National Institute of Health Research's School for Primary Care Research, aims to test whether using the MoleMate™ system (a novel device combining a diagnostic algorithm developed for general practice with SIAscopy, a multispectral skin imaging technique) will improve the effectiveness of management of suspicious pigmented lesions in primary care. For the trial, a 'suspicious pigmented lesion' is defined as any pigmented lesion presented by a patient, or opportunistically seen by a clinician, which cannot immediately be diagnosed as benign and the patient reassured.

The trial's pilot phase was set in 3 general practices in Cambridgeshire, and lasted 4 months. The entry criteria were patients aged 18 and over, presenting to their GP or practice nurse with a mole or other pigmented skin lesion which was not immediately diagnosed as benign. The only exclusion criterion was people considered by their GP to be unsuitable due to other on-going physical or psychological conditions. Ninety participants were recruited and, after completing their pilot trial consultation, all participants were then invited to be interviewed. Participants were interviewed within one month of their trial consultation and asked to reflect on the steps which led towards their presentation in primary care. A flexible interview guide was used to ensure consistency across the interviews, while allowing interviewees to express their ideas, understanding and concerns freely. This guide contained questions about knowledge of skin cancer and its risk factors, the circumstances leading to consultation, and the process of the consultation itself:

Why did you go to see your Doctor or Practice Nurse initially?

When did you (or another person) first notice your mole (the changes in your mole)? What did you notice?

What were your thoughts about the mole (the changes in your mole)?

What was different about it (compared to other moles)?

What explanation did you have for the changes?

What made you decide to see a health professional?

Did you talk with anyone else about your mole (changes in your mole)?

Did you think it could be serious?

Was your decision to consult the health professional influenced by anyone else?

How would you feel going back to your Doctor about other moles?

Could you tell me what you know about skin cancer? What causes do you know of? Where have you learned this?

We selected a convenience sampling strategy and the first 40 people to agree were interviewed: the data were then judged sufficient to reach saturation of the main themes. All participants were interviewed over the telephone by one of the authors (EH). The interviews lasted up to 25 minutes and were audio-taped and fully transcribed.

**Figure 1:**
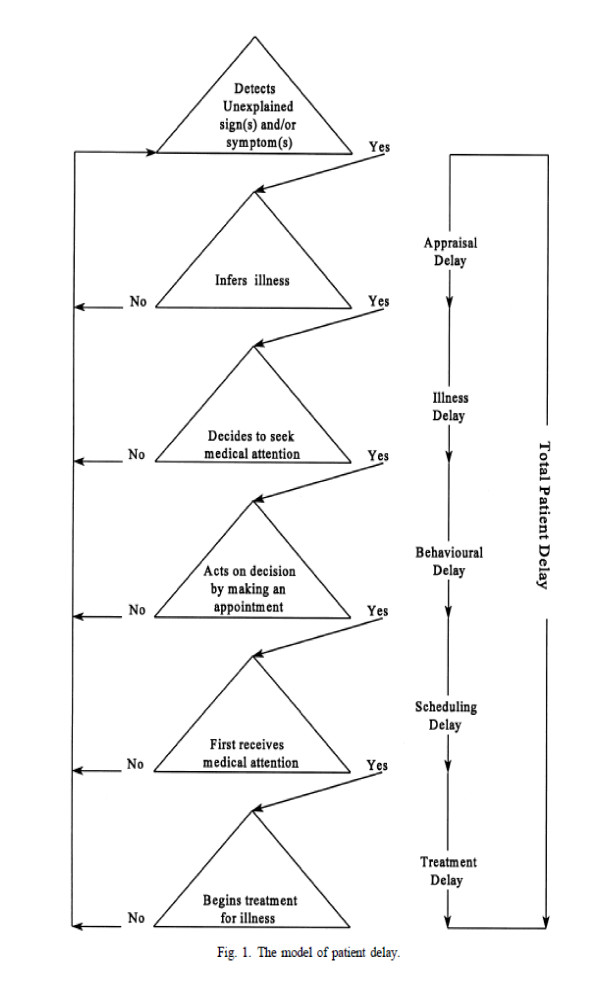
**The Model of Total Patient Delay, after Safer **[16]**and Andersen **[17].

In order to gain an overall appreciation of the interviewees' understanding, each transcript was read and reread by 2 authors (EH, FW), and this process of reading and comparison was used to identify themes [15]. About half of the transcripts were also read by the other members of the research team so that the analysis could be interpreted and refined though regular discussions. At this stage we incorporated the theoretical perspective of Safer and colleagues [16], modified by Andersen and colleagues in their model of Total Patient Delay [17], which gives detailed stages of a patient's pathway from awareness of a symptom to diagnosis and treatment. The model identifies 'appraisal, illness, behavioural, scheduling and treatment delays' referring to the time between a patient first detecting change, appraising change, inferring illness, deciding to consult, visiting primary care, and referral, diagnosis and commencement of treatment (see Figure 1).

We used the model's key stages as a framework to structure our coding, although we omitted consideration of referral, diagnosis and commencement of treatment issues as these were beyond the scope of this study. Transcripts were coded using the software package NVivo and five transcripts were independently coded by EH (a science graduate) and FW (a GP) who then further refined the coding frame with discussions about areas of disagreement and consensus. Data were then analysed using the widely used Framework approach [18]. Comparisons were made within interviews and across interviews to identify both patterns of themes and deviant cases. The quotations that follow were chosen to reflect a range of both consensual and dissenting views, and they are accompanied by the patient's study identification number, gender, age and educational level (EL1 = no qualifications; EL2 = GCSE or similar; EL3 = A Level or similar; EL4 = higher education or similar; EL5 = degree or similar). * denotes subsequent diagnosis of melanoma.

## Results

See Table 1 for characteristics of participants interviewed.

**Table 1:** Characteristics of participants interviewed (n = 40)

	Number or range
**Age**	
Mean	57 *
Range	22 - 83
	
**Gender**	
Female	25
Male	15
	
**Ethnic origin**	
White British	39
Chinese	1
	
**Employment status**	
Employed	18
Looking after home/family	1
Retired	17
Unknown	4
	
**Education level**	
No qualifications (EL1), GCSE (EL2), A Level (EL3)	17
Higher education (EL4), Degree (EL5)	21
Unknown	2
	
**Referral status**	
Not referred	24
Referred	16
Reassured	5
Biopsied and benign	7
Melanoma	4

* Mean age: Female 53, Male 63

The route to seeking advice about a mole or help with concerns about skin cancer symptoms was often complex and iterative. Some people seemed to have a clear idea of risk factors and symptoms of skin cancer, and for them the recognition of one of these prompted a decision to seek help. More people seemed much less well informed about changes in moles and possible symptoms of skin cancer. Using the framework of the Safer-Andersen model, we have characterised the factors influencing patient understanding and presentation in primary care as follows: pre-existing awareness, detecting and appraising change, inferring illness, and deciding to seek help.

### 1 Patient understanding

#### 1.1 Pre-existing awareness

In accounts of their pre-symptom experiences, people described knowledge of changes in shape, size and colour of a mole as possible skin cancer symptoms. Some also mentioned other symptoms such as oozing, bleeding and irritation. Only a minority appeared to apply this knowledge to themselves, as few linked changes in their own moles with skin cancer or the need for potential symptoms of melanoma to be seen and treated promptly.

*[Melanomas can] change in size, they itch... they change colour, they change shape, they can bleed and crack and just start doing things that they never did before*. [ID16: F, 61, EL1]

*Certain types of moles can create problems and if not nipped in the bud could spread nastily, that sort of thing*. [ID06: M, 67, EL5]

Most people were aware of sun exposure as a risk factor for skin cancer and some also mentioned the risk of sunburn, particularly as a child. Other risk factors such as skin types, sun bed use, and family history were discussed less often. Nevertheless, people who did mention family history as a risk factor appeared to be alert to changes in their own moles, and the possible significance of such changes.

*Well I worry about skin cancers because it's in the family, there's lots of cancer in the family and there has been some skin cancers, so I do worry about that*. [ID28: F, 38, EL3]

Personal meaning was given to people's knowledge about moles and skin cancer by their experiences not only with their own moles, but also those of significant others such as family members and friends. Most people felt that personal past experiences both heightened their awareness of new changes in their own moles, and 'allowed' them to seek further help. A few related that their past personal experiences, or the experiences of family or friends, made them feel much more vulnerable to skin cancer. Several people mentioned being a 'moley' person, or belonging to a 'moley family', but this did not seem directly linked to either feeling particularly protected from, or particularly vulnerable to, skin cancer.

*It then makes you more aware of the other moles, so... there's a terrible tendency to become slightly hypochondriac about it and think "hang on a minute, I think this other one looks a bit dodgy now"*. [ID09: M, 60, EL5]

*I've had a few friends who've had cancerous moles removed...so that makes you a little bit more aware that people of your age group can have it*. [ID24: F, 30, EL5]

*We are a 'moley' family- I have two on my stomach to the right of my umbilicus and my daughter has some in the same place and even my mother-in-law (laughs) which is a bit bizarre, and my mother*. [ID15: F, 61, EL5]

The impact of health promotional literature appeared to be limited, and many people were unaware of skin cancer preventive messages concerning use of suncream and sunbeds. One woman mentioned that the wording of a health promotional leaflet had more impact than the photographs which she felt did not resemble her own mole. In contrast, the impact of information from the non-medical media appeared to be more influential: people recalled reading about celebrities having skin cancer and newspaper articles about sun risks.

*I had picked up a leaflet in the local chemist and that did have pictures and diagrams about moles and when you should consult somebody...mine didn't look anything like the ones that were in the pictures, but in the descriptions it was talking about irregular [shapes] and bleeding and...it was those two elements that I picked up on*. [ID22: F, 41, EL5]

*I remember before I made the appointment I was reading something on John McCain, the US presidential candidate who has had skin cancer as well, and that article in the magazine made me make the appointment*. [ID40: F, 40, EL5]

#### 1.2 Detecting and appraising change

Most people seemed to hold some kind of an idea, or a mental model, of a 'normal' mole. When a particular mole was visible to them, people detected changes in the size, colour or shape, and became aware that one was different: this generally led to an appraisal of features which set it apart from the 'normal' mole. Many people appraised these changes by making comparisons with other 'normal' moles on their own body. Some people also compared their moles with the moles of other family members or friends, and a few people used health promotional literature or the non-medical media. Some people also appraised a change in their mole against their awareness of risk factors for skin cancer.

*This one is not an ordinary mole, it's not like the other moles I've got*. [ID04: F, 52, EL3]

*It just sort of struck me one day that it was darker than it used to be, that it was suddenly noticeable where it hadn't been previously*. [ID26: M, 22, EL5]

*The others don't bother me in the way that this one did because they just look like all the others, you know, perfectly round and brown and harmless.... I was concerned because it was raised, and it was also very dark and didn't seem to look like any other moles that I'd got*. [ID31: F, 52, EL5]

*I hadn't spotted it there before...and because it's on my shoulder and I quite often get sunburn on my shoulders, you know, I sort of figured it might be an idea to check it out*. [ID24: F, 30, EL5]

However, about half the interviewees had a lesion on an area of their body which was not easily visible to them, thus making it difficult to detect change. These people reported that their mole had been first noticed by another person, usually their partner or close family relative, and sometimes by a health professional.

*My wife told me that my mole on my abdomen had enlarged since she last saw it....well, you know how it is, you don't notice it for years on end and then I had no idea whether it's got bigger or not really*. [ID06: M, 67, EL5]

*[The consultation] was about this knee actually... I took my trousers off and this doctor came in and he called this other doctor in, and she said straightaway "How long have you had that mole?" *[ID19*: F, 79, EL1]

### 2 Presentation in primary care

#### 2.1 Inferring illness

About half the interviewees denied any concern about their moles and had not considered that the mole might signify an illness, either serious or trivial. These people had been persuaded to seek help by concerned partners or other family members, or had had a lesion noticed by a health professional during a consultation for another reason.

*Well funny enough, my son, he's only seven actually, and he'd mentioned it a couple of times "are you going to go and see the doctor with your mole?" and that's really what made me go in the end*. [ID12*: M, 61, EL3]

Among those who could see their own mole and had detected changes, people took a variable amount of time to appraise these changes and only some inferred illness. All interviewees discussed the implication of the size of their mole: the sizes mentioned ranged from 'tiny' to 'very small' to 'getting bigger'. A small size usually indicated a lack of seriousness, while an increase in size or changing shape seemed to indicate the potential for a more serious diagnosis. In particular, a combination of two or more changing symptoms contributed to the inference of illness and the decision to seek help. However, several people became concerned about the seriousness of the symptoms despite the mole continuing to have a 'small size' with no associated changes.

*It's not a big thing, it's very small... I can hardly notice it, and I've looked in the mirror, I can see it but it's only like a little pinball. It's not big...that's why I'm not too worried*. [ID23: M, 79, EL1]

*The one on my chest ... [is] the biggest, you know it's bigger than anything else I've got and it's quite red in colour so it did concern me*. [ID18: F, 44, EL5]

A few people mentioned symptoms other than visible or external symptoms. These 'internal' symptoms, such as lumps or pain, appeared to signify a more worrying bodily change with suggestions of 'invasion'. As people appraised the changes in their mole, an 'internal' symptom seemed to add gravity, and one elderly man described his lack of concern when noticing a mole getting bigger on his face as due to the fact that there was not also a lump:

*I've had it for years really, the mark in the middle of my forehead....It's actually got bigger itself on the forehead but it's still flat, it's not like a big lump or anything like that. [Lesion identified by Health Professional] *[ID07*: M, 83, EL unknown]

*I've had this mark on my leg for years...and just recently, within the last three or four months, it has started to pull, so the mark is on the, upper surface of my thigh, and it feels as if it's pulling down into the knee*. [ID15: F, 61, EL5]

Generally, there was a lack of connection between moles and skin cancer: among the people who had detected changes in their moles, many did not recognise the changes as possible symptoms of skin cancer. Conversely, there were many who did recognise possible symptoms of skin cancer, yet most did not seem to fear a 'serious' diagnosis.

*I don't quite understand...why a mole should then signify that you've got cancer or something like that, you know. Why is that? *[ID12*: M, 61, EL3]

*Well [it's] nothing really to worry about is it?...it's just a simple mole...I don't think that sort of cancer is life threatening..I mean...surely if that was going to kill me it would have done it by now...It's not a big thing.... He noticed this mole and he said it looked cancerous...I don't really know anything about it.. It's just a mole as far as I'm concerned*. [ID23: M, 79, EL1]

*I suppose I pushed it to the back of my mind really and didn't want to think it was serious. But there's always a niggling question over these things*. [ID18: F, 44, EL5]

#### 2.2 Deciding to seek help

Most participants who could see a change in their mole monitored and appraised it over a period of time ranging from a few weeks to several years before deciding to seek help. Rapid or multiple changes prompted people to seek help sooner, while gradual or 'steady' change did not prompt people to seek help.

*After about 3 months [I thought] maybe I ought to do something about it and then after a period of another week or 2 I decided to make an appointment... it was getting slightly bigger and slightly darker all the time ... it's gone from nothing at all ...so gradually it was getting more prominent*. [ID09: M, 60, EL5]

*I think I'd caught it early enough on, so that if it did prove to be something nasty then I'd hope that I was in plenty of time for it to be removed*. ID28: F, 38, EL3]

Deciding to seek help also depended on access to primary care professionals: while some people felt access was readily available, others had concerns about encounters with primary care professionals. We identified three issues which seemed specific to people seeking help for their moles.

First, some people expressed the need to have their concerns 'taken seriously'. They mentioned a range of factors which might lead to not being taken seriously, including being considered to have wasted their doctor's time, or consulted with trivial symptoms or unnecessary worries.

*In my opinion the medical profession has a tendency to think, If [the reason] is not important, why should people be worrying about it. Well you know, anybody's worry is very significant to them even if it's totally unfounded*. [ID09: M, 60, EL5]

*I brought up the issue of there being lots of cancers in my family and I feel at that point then it was taken a little bit more seriously*. [ID28: F, 38, EL3]

Second, many people felt they needed to justify (either to themselves or the GP) their decision to see their doctor, particularly if the mole was small. Some felt that monitoring their lesion over time provided enough justification, while others needed to accumulate more than one reason to consult:

*I left it a couple of weeks and I just thought well, you know, I've got to be sensible about this, rather than wasting doctor's time, I just thought it was something I should follow up on*. [ID34: F, 54, EL2]

*It would have been several weeks, it might have been even a couple of months...and I think when I went to see him I actually went about something else as well...Well by the time I'd accumulated two or three things, I felt justified to visit*. [ID31: F, 52, EL5]

Third, people also often needed to have a catalyst or trigger to seek help: this could include noticing a new change in the lesion or a combination of symptoms, or perhaps an observation made by a family member or friend.

*I had one on my tummy, probably about 5 months ago, and I kept thinking "I must go and talk to the doctor about that, I must go and talk to the doctor about them". And then I found another one just sort of between my armpit and the side of my breast and then when I noticed that, and when it bled I went and saw the doctor ... about a week and a half later*. [ID22: F, 41, EL5]

*I knew I had this mole on the back of my arm...And people said oh you ought to get that checked out and I kept forgetting. And I work...at [the hospital]...and one of the nurses said have you had that checked out, so I made an appointment to go the doctors and I asked her to have a look at it*. [ID36: F, 46, EL4]

### 3 Factors influencing presentation in primary care with melanoma

Four interviewees went on to have melanoma diagnosed and treated. The patient characteristics and accounts of their pre-presentation experiences were compared with the characteristics and accounts of those diagnosed with benign lesions.

All four people with a subsequent melanoma diagnosis were older than the mean age (83, 83, 79 and 61), and three were male. They had a range of educational levels, from no qualifications to a degree.

Table 2 highlights factors in the four interviewees' accounts of their pre-presentation experiences. All gave some indication of a pre-existing awareness of skin cancer symptoms and the risks of sun exposure. Three had either lived in tropical climates as a child or adult, or had an above-average exposure to sunlight due to their occupation. Two gave accounts of friends or family members undergoing recent treatment for skin cancer, and one recounted a personal experience of mole removal in the past. All had been aware of their pigmented skin lesion for a long while, ranging from months to years. Two had noticed changes in either size or colour, and one recollected, with his partner, comparing the changing mole with a description in a paper and both concluding that the changes were not serious. None admitted to inferring illness or having any concerns that the changes could be serious. One felt that this was because the lesion was flat rather than lumpy, and one gave a range of reasons including that the lesion was not easily visible, that he was not systemically unwell, and that his business commitments were more important than an appointment with his doctor. Despite all having some pre-existing awareness of moles and skin cancer, and most having detected changes in their mole, three out of the four had their suspicious lesions incidentally noticed by their doctor during a consultation for another ailment rather than deciding to seek help. The fourth had been persuaded to seek help by his worried young son.

**Table 2:** Factors affecting presentation of people subsequently diagnosed with melanoma to their GP

	Pre-existing awareness	Detecting and appraising change	Inferring illness	Deciding to seek help
**Patient ID 5** [M, 83, EL 5]	*Grew up in hot climate and over-exposed to sunlight *Several moles removed some years ago *Friend and daughter recently treated for skin cancer	*Aware of mole for about 2 years	*'I thought it was something else'	*Noticed by GP *Felt 'alarm, and relieved that it had been noticed'.
**Patient ID 7** [M, 83, EL unknown]	*Worked outdoors 'all adult life, often with shirt off, which I regret now' *Many black moles on back	*Aware of mole in middle of forehead for 'a long while' 'had it for years' *New black mark in centre *'Its got bigger'	*'I kept thinking it was a birthmark' *It's still flat, not a lump'	*Noticed by GP
**Patient ID 12** [M, 61, EL3]	*Aware of danger of sun damage *Friend recently treated for skin cancer on face	*Aware of mole going darker 'months ago' *Compared mole with description of melanoma in paper but he and partner felt not similar	*Not easily visible so not concerned *Competing demands of self employment *Able to play golf so 'How can I be ill?'	*Young son suggested visit to doctor 'a couple of times' before made appointment
**Patient ID 19** [F, 79, EL 1]	*Lived in tropics for many years as adult *Wears suncream to play golf	*Noticed adjacent mole which is 'bigger and darker' about 1 year ago		*Noticed by GP

## Discussion

Our data illuminate patient understanding of moles and skin cancer, as well as the stages people go through from noticing a change in a mole to presenting in primary care with a suspicious pigmented skin lesion. Key findings include: the important role that personal, family and friends' experiences of moles and skin cancer can take in informing not only knowledge and understanding but also the appraisal of lesions and seeking help; using comparisons with moles on self, other people or pictures to assess changes; the role of family, friends and health professionals to detect and appraise changes in less visible lesions; and the role of multiple symptoms, a lump, or pain, in heralding seriousness. It is noteworthy that up to half of the interviewees said they were not concerned about their moles. We presented the four patients subsequently diagnosed with melanoma as case studies to illustrate that, despite national health promotion strategies such as SunSmart, these people did not recognise melanoma symptoms or associate risk factors such as excessive sun exposure with themselves.

### Strengths and weaknesses of the study

The two key strengths of this qualitative study are that it addresses an important clinical area where the evidence base is weak, and it applies a theoretical model to underpin the analysis. Other methodological strengths included the size of the sample, and that four of these forty participants went on to have melanomas diagnosed.

While we acknowledge that data are drawn only from people who were recruited to the MoleMate™ UK Trial and that the greater majority of these people had a benign lesion with only a small minority subsequently diagnosed with melanoma, these interviews with trial participants, conducted as soon after their trial consultation as possible, achieved fresh accounts of the pre-presentation and diagnostic period. Other studies have reported retrospective narrative interviews [19-21]: these have mainly been conducted some time after the diagnosis of melanoma and the accounts may not accurately relate what really happened as they rely on people's recall of events [22]. This qualitative study aimed to describe a range of patient views rather than numerical representation and generalisibility. We therefore included people with a wide range of experiences and background. Ethnic variation was limited, reflecting the geographical region in which the study was carried out. We acknowledge that the interviews were brief, and suggest this was due to the practical issue of using telephone rather than face to face interviews. Face to face interviews might have allowed the exploration of peoples' understanding and experiences in more detail, but this approach did not appear to compromise the quality of the data. We also acknowledge the limitation of recruiting from only 3 practices in Cambridgeshire: while this limits the generalisibity of the findings, we found most beliefs and understanding to be widely held. It may be that understanding of moles, skin cancer and seeking help may vary across the UK, countries, and health service systems.

### Using the theoretical Model of Total Patient Delay

Our findings suggest that the stages are not always passed through successively and independently as suggested by Andersen *et al*., but that the stages can be merged or omitted altogether (for example, one participants subsequently diagnosed with melanoma was aware of changes in his mole, but had decided not to seek help as he did not feel unwell). This suggests that while the model provides a useful description of the process, the stages do not necessarily exist in the mind of many patients, with important implications for research which aims to use the stages of the model to determine the interval lengths from symptom awareness to diagnosis. We have also highlighted novel themes such as a pre-existing awareness of moles and risk factors for skin cancer. We tentatively suggest that the model, which currently includes appraisal, illness, behavioural and scheduling delay intervals, could be expanded to include the stage prior to the appearance of symptoms, and could also be broadened to include the significant role of others. We are also concerned that the model implies that all individuals are 'delayers', as this term is value-laden and may imply negative outcomes and fault. In reality, patients are frequently involved in a variety of judgements and reflections prior to seeking attention that do not correspond with a simple description of barriers or promoters for seeking help. We would therefore suggest replacing 'delay' with 'time to presentation' wherever possible.

### Clinical implications

Diagnosing melanoma is a challenge in primary care [23] as people present frequently with moles and other pigmented lesions, yet the incidence of melanoma is low: a GP may diagnose melanoma in only one patient every three to five years. In our study three of the four subsequently diagnosed melanomas were opportunistically noticed by the GP during a consultation for another problem, a higher proportion than in other studies which suggest the majority of melanomas are self-discovered [24]. Despite this, many people mentioned the GP as a barrier to seeking help. Similar views have been expressed in other studies of people with cancer [25], and are important as experts have questioned whether the 'GP as gatekeeper' model of health care has contributed to lower cancer survival rates found in the UK compared with other Western European countries [26]. To overcome this potential barrier, GPs can encourage people to mention all their concerns at the beginning of a consultation, and then triage the list according to the level of concern of both patient and doctor. Internet-based diagnostic tools such as self assessment algorithms may provide more means for patients to overcome these barriers in the future.

The findings from the study also have implications for patients, as the participants frequently did not consider their changing mole to be symptomatic of melanoma. This may be due to the perceived trivial nature of the symptoms: people with cancer of the skin, mouth and cervix have been found to be less likely than those with breast cancer to seek help [27]. Some participants misattributed changes in pigmented lesions when visible, or were unaware of changes when not easily visible to themselves. A UK primary care population has been shown to be more concerned about bleeding, oozing and crusting as symptoms of melanoma, rather than changes in shape or colour in pre-existing moles [28], compared with a similar Australian population who were better informed about the importance of change in a pre-existing lesion [29]. Lay understanding may also vary between genders, ages, ethnic groups and educational levels [30]. The importance of other people along the patient's pathway towards seeking help was also highlighted in this study. Whilst on some occasions the importance of relatives or friends was pragmatic, for example to see a mole on the back that a patient couldn't see themselves, more generally it was through the dialogue with others that previous experiences and alternative perceptions of the mole and its possible changes were discussed, and led people to finally seek help.

Finally, the study has implications for public health strategies such as SunSmart, as many participants were unaware of sensible sun-exposure behaviours, and educational strategies to promote skin awareness, self-examination and timely consultation. Our data suggests several key areas which are important for future research, and likely to provide significant resources for successful health promotion. First, we need to create a stronger link between people's mental models of moles and melanoma for effective symptom appraisal. Health promotional images may be more useful if they reflect a broad range of melanomas, particularly at earlier stages. Second, images may be enhanced by written descriptions of early changes, and patients' narratives to encourage people to develop personal meaning from knowledge (see DIPEx Health Experiences Research Group, http://www.primarycare.ox.ac.uk/research/dipex/). Third, promoting knowledge about 'normal' vs 'abnormal' moles may be helpful, as well as challenging incorrect beliefs such as a mole needing to become lumpy or painful before it may be considered 'serious'. Such knowledge could be promoted alongside celebrity accounts as the lay media appeared to be far more influential than the medical media. Finally, future awareness campaigns could be targeted at the higher risk groups, such as elderly men or people living alone, and they could promote the responsibility of family and friends in appraising changes in moles and reducing delays in seeking help.

## Conclusions

This study has identified factors which influence patient understanding of moles and skin cancer, and that can trigger or act as barriers to seeking help for suspicious skin lesions in primary care. Skin lesions are often felt to be trivial, and changes may not signify possible skin cancer. Further research relating to patient understanding of information and images of moles and melanomas could improve appraisal delay, and contribute to the current national strategy to improve patient awareness and earlier diagnosis of cancer.

## Competing interests

The authors declare that they have no competing interests.

## Authors' contributions

EH interviewed the patients and analysed the data with FW: all authors contributed to the regular analysis consensus meetings. FW drafted the paper. All authors contributed to subsequent drafts and all read and approved the final manuscript.

## Pre-publication history

The pre-publication history for this paper can be accessed here:

http://www.biomedcentral.com/1471-2296/11/62/prepub
